# Exploring trauma associated appraisals in trauma survivors from collectivistic cultures

**DOI:** 10.1186/s40064-016-3043-2

**Published:** 2016-09-15

**Authors:** Alberta Engelbrecht, Laura Jobson

**Affiliations:** 1 Department of Psychological Medicine, Institute of Psychiatry, Psychology and Neuroscience, Kings College London, London, UK; 2 Monash University, Melbourne, Australia

**Keywords:** Appraisals, Culture, PTSD, Qualitative, Trauma

## Abstract

Appraisals are a key feature in understanding an individual’s experience; this is especially important when the experience is a traumatic one. However, research is diminutive when looking at the interaction between trauma appraisals and culture in relation to posttraumatic stress disorder using qualitative methodologies. This study explored cultural differences in perceptions and appraisals of trauma using three qualitative focus groups with community members (n = 11) from collectivistic cultures who had experienced a traumatic event and three qualitative individual key informant interviews with mental health practitioners (n = 3) routinely working with trauma survivors. Using template analysis, eight emergent themes were highlighted from the data sets [(1) trauma and adjustment; (2) cultural and social roles; (3) traumatised self; (4) relationships; (5) external attribution; (6) future; (7) education; (8) language] that potentially have significant consequences for posttrauma psychological adjustment and recovery. Cumulatively, while a number of themes are similar to that which is emphasised in current literature (e.g. damaged self, negative appraisals of the world, others, future) a number of themes were also resonant and warrant further scrutiny. For instance, the importance and interconnectedness of the group to the individual and the impact trauma has on this; the importance of social roles, cultural appropriateness and violations of cultural values and norms; findings and implications are discussed.

## Background

Appraisals are a key feature in understanding an individual’s experience; this is especially important when the experience is a traumatic one. Appraisals enable an individual to derive or construct meaning from a traumatic event that is potentially meaningless and arbitrary. Thus the manner in which the trauma is appraised is of paramount importance as it allows for evaluation of how the individual navigates through a series of novel and unwanted experiences, thoughts, emotions and behaviors. Appraisals aid in the understanding of posttraumatic psychological adjustment and recovery, along with what impedes its progress (e.g. Kleim et al. 2007).

Posttraumatic stress disorder (PTSD) has received substantial clinical and empirical focus in the last two decades. Much of the literature on trauma and posttraumatic stress is based on cognitive models of PTSD, which emphasize the effects of trauma on the primary victim (i.e. the individual directly experiencing the traumatic event) (Brewin and Holmes 2003; Ehlers and Clark 2000). However, it can be argued that the culture in which the primary victim(s) are oriented in plays a crucial role in their experiencing and appraisals of the trauma event. Shaler (2005) supports this assertion and purports traumatic events to be perceived as traumatic when it is both emotionally and personally meaningful. Moreover, Shaler (2005) puts forth that a traumatic event should not be viewed as affecting individuals; instead it should be viewed as affecting humans in their context. One’s culture provides such a context, namely the context in which humans reside, from which they draw meaning, and determines whether particular explanations, appraisals and cognitions make sense. Thus, following traumatic events, these cognitive understandings of the world are called into question and have the potential to have extremely detrimental effects on an individual; namely PTSD (Ehlers and Clark 2000). Also, and importantly, culture influences how others within one’s culture would appraise the traumatic event and a trauma victim’s response posttrauma. This potentially affects an individual’s support system, either enabling or disenabling it at the group level. Thus, the relationship between trauma and culture is an important one and warrants further investigation for several reasons; not least of which is to arrive at culturally informed and appropriate PTSD models and treatments for those who have experienced trauma from non-Western, cultures (Engelbrecht and Jobson 2014; Jobson 2009; Jobson and O’Kearney 2006, 2009).

Culture influences how individuals think, feel and behave (Ford and Mauss 2015). The framework most often used to characterise this influence focuses on the extent to which a culture promotes *interdependence* versus *independence* (Markus and Kitayama 1991, 2010). Specifically, people in different cultures have strikingly different understandings of the self. In individualistic cultures (typically Western) the self is perceived to be an independent, autonomous and self-determining unit. In contrast, in collectivistic cultures (typically non-Western) the self is perceived as an interdependent, related unit (Hofstede 1980; Hofstede and Hofstede 2004; Markus and Kitayama 2010). Such culturally diverging self-construals have been found to impact on, and in many circumstances govern, the very nature of individual experience, including appraisals. Cultural differences in self-construal are shown to impact on the way in which events, situations, and life encounters are appraised (Mesquita and Walker 2003). Markus and Kitayama (1991) highlight that individualistic cultures appraise success through independent, personal accomplishment and a personal sense of control, while in collectivistic cultures, “agency is differently instantiated…or is not valued as much” (Mesquita and Walker 2003, p.785) but rather fate, adjustment to the situation, and the interdependence of an individual and their social environment are stressed (Mesquita and Walker 2003). There is considerable support for these notions in terms of everyday experiences (Mesquita and Walker 2003). It is important to note that variation exists in the degree to which individuals exhibit an independent versus interdependent orientation both within and between collectivistic and individualistic cultures; however, normative differences between collectivistic and individualistic cultures are marked (e.g. Fiske et al. 1998).Despite this substantial body of cross-cultural literature examining appraisals of life experiences, to date, little research has investigated the influence of cultural differences in self-construal on trauma-related appraisals in trauma survivors from non-Western, collectivistic societies (Engelbrecht and Jobson 2015).

There has been much quantitative and empirical research delving into the impact that trauma appraisals have on the development and maintenance of PTSD. However, the same cannot be said from a qualitative viewpoint. There are very few published studies addressing trauma appraisals using qualitative methodologies. Research is even more diminutive when looking at the interaction between trauma appraisals and culture in relation to PTSD. Therefore, the use of qualitative methodologies to understand the interplay of culture and trauma (i.e. trauma appraisals) is important as it could provide valuable information to improve standards of care and access to services for those from non-Western populations. This study therefore aimed to help bridge this gap. To accomplish this, the study focused on exploring the perceptions, understandings and interpretations of trauma survivors from different collectivistic cultural groups through the use of focus groups and interviews, as these are particularly sensitive to cultural variables and highlight the dynamic nature of people’s understandings (Kitzinger 1994). Participant selection was based on community members from collectivistic cultures who have experienced a trauma and mental health practitioners who specialize in working with trauma survivors from collectivistic cultures. This provided multiple perspectives, which are essential if insights into questions exploring this topic are to be gained.

Therefore, in order to gain further understanding of cultural differences in perceptions and appraisals of trauma, the study aimed to (a) investigate what meaning(s) community members from collectivistic cultures attach to trauma (and in particular appraisals typically generated in such groups) and whether this is influenced by culture; and (b) use key informant interviews to further elicit insights on the influence culture has on trauma appraisals within collectivistic cultures.

## Methods

### Design

The study used three qualitative focus groups with community members from collectivistic cultures and three qualitative individual key informant interviews with mental health practitioners. This number was deemed appropriate as participants had unique knowledge and experiences to draw upon to shed light on the topic being investigated (Fern 2001; Krueger and Casey 2000; Merton and Kendall 1946; Morgan 1997). To provide context for the research process, it is important to note that data analysis and reporting was conducted using a team-based approach. The team consisted of a primary (A.E) and secondary (L.J) investigator. This approach aided the systematic coding of text, allowing for open dialogue between the investigators in regards to defining the multiple interpretations of codes and thematic developments and acting as an inter-rater agreement measure.

### Data collection and measures

Semi-structured, in-depth interviews were used to enable a detailed exploration of trauma appraisals from a collectivistic sample’s views, perspectives and experiences. The questions were based on previous research which had demonstrated that following a trauma, appraisals or re-appraisals of the self, world and others are frequent and key in influencing cognitive appraisals related to the development and maintenance of PTSD (e.g., Ehlers and Clark 2000; Foa et al. 1999). Participants were asked 1. What does trauma mean in your culture? (Focus group)/What does trauma mean in collectivistic/interdependent cultures? (Key informant interviews); 2. What typical thoughts do people have after a trauma? 2.1. About themselves? 2.2. About the world in which they live? 2.3. About their future? 2.4. About their relationships with others? and lastly 3. How do these thoughts influence adjustment?

All focus group sessions and key informant interviews were audio taped, transcribed verbatim and checked for accuracy. The moderator (A.E.) also took notes on interpersonal dynamics and nonverbal communication among participants that could not be captured by audio recording (Kitzinger 1995; Krueger and Casey 2000). The transcripts revealed that at times the moderator asked follow-up questions that were subsidy to the topic guide in order to clarify a response or encourage participants to provide more information on the general topic questions put forth.

### Procedure

Ethical approval for the study was obtained from NRES Committee East of England REC (reference number 10/H0311/56).

### Focus groups

All participants (*N* = 11; male *n* = 8, female *n* = 3) for the focus group were recruited from the general community in Norwich by using posters at a non-profit charity based in Norwich and internal bulletins to its members and affiliates. Notices called for participants who were from collectivistic communities, were aged over 18 years and who could complete the study in English. Those who contacted the researcher were invited to the focus groups. Potential participants for the focus groups were randomly allocated to Focus Group 1 (*n* = 4), Focus Group 2 (*n* = 4), or Focus Group 3 (*n* = 3). Focus groups were run on separate days. Participants were informed about the study’s purpose, limits of confidentiality and the right to withdraw. Following written informed consent procedures, participants commenced the focus group sessions, which lasted approximately 1 h. The focus groups took place in a pre-booked room at the university and were led by the primary investigator (moderator, A.E.). At the start of the focus groups and prior to audio recording, participants introduced themselves to each other, and disclosed demographic information pertaining to their ethnic identification, age, employment, education and time in the UK (see Table 1). After introductions were made, the moderator called the focus group to a start. At the end of the study participants spoke privately with the moderator and disclosed their trauma event and any thoughts they had regarding the study. At the end of the focus group session participants were given £15 to compensate them for their time.

**Table 1:** Focus group and key informant demographic information

Focus group no	Participant number	Age	Gender	Ethnicity	Trauma event	Education	Residency status in UK and employment status	Time in UK	Collectivism (Hofstede and Hofstede 2004)
*Focus group*									
1	P1	25	Female	Jordan	RTA	Masters	International Student status	<1 year	38
1	P2	29	Male	Indian	RTA	Masters	International student status	1 year	48
1	P3	28	Female	Slovakian	RTA	Masters	International Student status	2 years	52
1	P4	20	Female	Chinese	RTA	BSc	International student status	2 years	20
2	P5	28	Male	Indian	RTA	Masters	International Student status	<1 year	48
2	P6	26	Male	Sri Lankan	Witness death	Masters	International student status	<1 year	–^a^
2	P7	26	Male	Sri Lankan	RTA	Masters	International student status	<1 year	–
2	P8	27	Male	Sri Lankan	RTA	Masters	International student status	<1 year	–
3	P9	22	Male	Chinese	Accident/injury	Masters	International student status	1 year	20
3	P10	23	Male	Vietnamese	RTA	Masters	International student status	1 year	20
3	P11	29	Male	Ethiopian	Persecution	A-Levels	International student status	<1 year	20

Interview session	Participant number	Age	Gender	Ethnicity	Type of trauma work	Education	Work experience	Time in UK	Collectivism (Hofstede and Hofstede 2004)
*Key informant interview*									
A	PA	45	Male	British	Community violence; domestic violence; refugee trauma	Ph.D.	Clinical psychotherapist; >10 years	45	89
B	PB	40	Female	British	Refugee and asylum seeker trauma; sexual abuse; domestic violence	Ph.D.	Clinical psychologist; >10 years	40	89
C	PC	40	Male	British	Refugee and asylum seeker trauma; community violence; domestic violence	Ph.D.	Counseling psychologist; >10 years	40	89

*RTA* road traffic accident

^a^Sri Lanka has not been given a collectivism score as Hofstede and Hofstede (2004) have done for the other countries. However, Sri Lanka is considered a collectivistic culture (Hofstede and Hofstede 2004)

Focus group participants were Chinese (*n* = 2), Vietnamese (*n* = 1), Indian (*n* = 2), Sri Lankan (*n* = 3), Ethiopian (*n* = 1), Jordanian (*n* = 1) and Slovakian (*n* = 1). All identified as being from non-Western cultures. According to Hofstede’s and Hofstede’s (2004) Individualistic/Collectivism continuum, all these nationalities fell within the collectivistic range 20–52. Focus group participants ranged in age from 20 to 29 years; all participants were unemployed and enrolled in higher education courses. Participants were all international students, living and studying in the United Kingdom (UK) between 1 and 2 years, for the purposes of their academic pursuits and all had International Student UK residency status. None of the participants were in employment of any kind and none disclosed themselves as refugees or asylum seekers. All participants identified as being trauma survivors and had a range of trauma experiences that occurred in the participant’s country of origin and prior to arriving in the UK (see Table 1).

#### Key informant interviews

Eight mental health professionals, who were identified^1^ by the researchers as having significant experience in this area (e.g., mental health practitioners working with refugees and asylum seekers and mental health practitioners working with immigrants who had experienced trauma), were contacted directly by email and invited to take part. Respondents asked for further information about the study. Three of the invited professionals consented to taking part in the study and were selected on the criteria that they had extensive experience of working with trauma survivors from a wide range of countries and cultures, including individuals from countries that would be considered as collectivistic on the Hofstede and Hofstede’s (2004) Individualistic/Collectivism continuum. The remaining five invited professionals did not take part as some declined due to time constraints, logistic impracticalities or that they felt they did not have the requisite experience of working with trauma survivors. Interviews were conducted in the interviewee’s offices. The interview commenced following the informed consent protocol. The interviews lasted approximately 1 h and were guided by the same open-ended questions.

The mental health practitioners all identified as British which amounts to a score of 89 on Hofstede and Hofstede’s (2004) Individualistic/Collectivism continuum. The age range of participants was between 40 and 45 years. All participants reported having a wide range of experience working in a variety of mental health settings that specialised in treating trauma survivors. These included, but were not limited to, domestic and community violence, armed conflict, and refugee and asylum seeker traumas which included exposure to conflict, torture and persecution.

All focus group and interview sessions were audio-recorded in order to transcribe verbatim and check for accuracy. Participants were notified of this from the start, prior to filling out consent forms. At the end of transcription, all recordings were destroyed.

### Data analysis

Data analysis utilized Template Analysis to code the focus groups and key informant interviews. This is a particularly apt method of analysis because it has been designed to analyze textual data including responses to open-ended questions as employed in this study (King 2008). Template Analysis allowed for the narrowing of extensive information captured by the focus group and key informant interviews. It further allowed for the development of a coding template, which was used to summarize themes, focused on patterns formed by words, themes and perspectives that emerged throughout the sessions, along with being able to organize them in a meaningful and useful way (King 2008).

#### Development of the template

*Step 1* A priori themes were developed. These were based on the interview questions and prior research which delineates that negative changes to views of the self and others, world perception, and future perceptions to be predictive of PTSD maintenance (Ehlers and Clark 2000; Foa et al. 1999). The initial themes were (a) traumatized self, (b) altered perceptions to worldview, (c) changes to future, and (d) dysfunctional relationships. *Step 2* Interviews and focus groups were then transcribed and read through for familiarization. *Step 3* Initial coding was carried out on the first focus group. The parts of the data that were relevant to the research questions were identified when they fell within the scope of the a priori theme. A code was then designated to this section of the transcript. While reading the transcript if there was no relevant theme that fit the section of textual data, a new theme was devised. Additional themes added at this point were: (a) trauma perceptions, (b) trauma symptoms, (c) cultural and social roles, and (d) external attributions. *Step 4* Initial coding was then carried out on all transcripts. During this process, identified themes were grouped into a smaller number of higher order codes, which described the broader theme in the data. This led to the initial template. *Step 5* Applying the initial template to the full data set developed the final template. Whenever a relevant piece of text did not fit with the existing themes a change to the template was needed. This was achieved through (a) emergent themes in the data that were not anticipated, (b) adding new codes to reflect these themes, (c) restructuring how the different codes fit together, and (d) deleting a theme because it was better covered by another. *Step 6* Final template is used on the full data set to interpret findings. *Step 7* At stages 4 and 5 a quality check was taken to ensure analysis was not being distorted by preconceptions and assumptions. This was achieved through independent scrutiny of the analysis by the other member of the research team, as detailed above, to ensure reliability and trustworthiness.

### Reliability

For the research design and analysis stage there were two checks of reliability and validity. First, a topic guide was used to ensure a similar range of topics was discussed with each participant. Second, the formal analysis and development of taxonomy was completed by the primary researcher (A.E); additionally, some of the transcripts were coded by a second rater (L.J) to ensure trustworthiness. Discrepancies between raters were resolved through discussion before arriving at a final coding framework. Additionally, although there was only a small number of focus groups and key informant interviews data saturation (i.e. where no new themes were emerging) was achieved after the first two focus groups and confirmed with the final focus group. Similarly, data saturation was achieved after the first two key informant interviews and confirmed with the final key informant interview.

## Results

The final Template identified eight emergent themes within the qualitative data sets: (1) trauma and adjustment; (2) cultural and social roles; (3) traumatized self; (4) relationships/others; (5) external attribution; (6) future; (7) education; and (8) language. To provide an understanding of our coding and interpretation of data, the findings are presented according to analytical typologies. Verbatim quotes from the study are from the focus groups or key informant interviews identified by participant number (e.g. P1). The first four themes are the most salient and pervasive, often overlapping with each other.

### Trauma and adjustment

Throughout the focus group sessions, trauma was predominantly thought of in terms of physical health while mental health or psychological health was not appraised to be of equal importance. “*Normally we never mention this word [trauma]… its not concerned with the mental, it’s from the outside … the body” (P4).* Additionally, the impact of trauma was discussed as posttraumatic somatic symptoms *“they can’t have a good sleep and they can’t eat anything”* (P2).

Subsequently, key informant interviews put great importance on meanings of trauma in collectivistic cultures as affecting the group and relationships within that group. Key informant interviewees denoted that trauma is experienced in an interdependent manner, as the rupturing of social and interpersonal bonds:

- *“if you’re from a collectivistic culture then bonds are everything, so its [trauma] something which break the family, breaks relationships, breaks your bond to society”* (PB]. Additionally, the interviewees reported that trauma is often “*experienced at the group level*” (PC) and is explained as a collective experience *“what’s important is what happened to the group and how the group responded, the family, the party, the village, the town, or whatever it is, the [group] as a whole”* (PC).

This theme continues in regards to adjustment, namely adjustment being interconnected with group support. As one key informant noted, *“The community… are extraordinary in how they look after each other”* (PC). Another key informant explained that *“people draw strength from getting support from other, feeling that they belong within a group, erm, feeling that they’re being helped and supported, and that gives them the motivation to put things right and to start you know trying to rebuild, er, it also helps them with the grieving process”* (PA).

### Social and cultural roles

Participants emphasized societal and cultural impact factors and endorsed the importance of social and cultural expectations and roles. For instance, following a trauma, *“it’s not only your own expectations but it what other people expect from you”* (P1). As one participant denotes *“[there are] expectations from society on you … you are expected to behave in some way and you sometimes are afraid of doing something different”* (P3). When these expectations are not met, the individual feels traumatized because they are in direct conflict with these expectations.

Moreover, from the responses it seemed that one’s culture assigns roles to its members; when individuals experience a trauma these social and cultural roles (e.g. I am a father/provider/caregiver) undergo change that can result in loss or damage to the self. For instance, individuals *“don’t say I’ve lost my role but men [as an example] … one way or another you’re led to a thought that this is somebody who had a place in the family, a place in society, erm, had a role who now doesn’t. So you know, going from provider and head of the family to being the one who is looked after because … he’s traumatized”* (PB). Thus when the self is thought of in terms of failure, or rather failing in its role, it can have very negative connotations, individuals *“think of themselves as very weak and not very strong to face these problems [resultant from the trauma event]”* (P6).

If individuals feel they have adhered to cultural and societal expectations and acted appropriately, yet still experience a trauma, they may see themselves as *“being cruelly marked out … why me? … I’ve done everything right, … I’ve been a good citizen, I’ve followed my religion, I’ve fitted in, I’ve been a good citizen, why has this happened?”* (PA). The protective features of one’s culture has not shielded the individual from suffering or pain, it has failed them, and subsequently challenged their beliefs, rendering previously held schemas for safety, trust and dependency as redundant and/or contested.

### Traumatised self

#### In relation to others

Those from collectivistic cultures were found to be concerned about others over and above themselves, whereby the family or the group is their raison d’être. They would sacrifice the self for the group, *“because if you have … a sense of a bigger group, then the bigger group can survive even without you”* (PB). Whereby, the individual is no longer the focus, they are not thinking of themselves, their individual future is over but their family’s future won’t be if they sacrifice their needs: *“I don’t care for myself but I’ve got children now”* (PB).

#### Self-blame

All study participants mentioned being highly emotive with appraisals centering around self-blame and feelings of guilt and shame; again all of which appear to be interconnected with others and one’s wider community.

*Example of guilt feelings:*

- *“*The event happened because of the way I acted, you hear that a lot, unrealistic guilt. We had a client who had been beaten unconscious by a group of soldiers who attacked his family and then his mother was killed, so he was actually unconscious at the time she was killed, so there was nothing he could have done and he was wracked by guilt*”* (PC).

*Example of feelings of shame:*

- “I’m thinking about young women who I’ve worked with over the years, who have been raped … shame coming into this at quite a communal level as an example … [abduction of women community assumes] you would have been raped. And so what we discovered was happening, was that then because you had been raped shame falls on you and your family erm and women would talk to us about feelings that they were impure, they were never able to get married erm and as we got into it more and more we discovered that actually what would often happen is that they would have to flee for their own safety and their family would have to entirely relocate because of the sense of shame” (PC).

### Relationships/others

Participants placed emphasis on others and the group they felt they belonged to, and focused on the importance of their relationships with them. Study participants found it very difficult to talk about themselves following a trauma. Instead they continually brought the conversation back to others and their relationship to others, in addition to the challenges and changes a trauma could play in their relationships to their family and community at large. Again demonstrating the pervasiveness of one’s group/collective.

Further, as highlighted with the 3 themes above, the group was suggested to protect, motivate, support and help, *“[for] recovery, I think the help from the family is very important … the family have the sole responsibility to the people who have the problem”* (P11). Conversely, the groups can also exacerbate problems the individuals face by judging and pressurizing them:

- “The culture in which they reside can also place greater strain on the individual, I work with people here, who talk about being blamed and judged by members of their community because of their misfortune, they’ve been tortured, or somebody’s been killed, or whatever, they’ve been blamed for it, because of what they did in a previous life” (PC)

### External causes

#### Fate

Fate attribution came up in all focus group session. Several respondents believed trauma events to be arbitrary and random, not necessarily brought on by anything the individual may have done but rather based the causality of trauma as a result of fate. As P4 denotes, *“sometimes we think fate [must have caused the event to happen], and there was a reason so … it must be something you had to experience*”.

#### Religion

Religion was another subtheme. For instance, P1 asserts, *“in my culture, my religion says that everything has happened is a plan from God and it’s kind of a test”.* Participants reported that if a trauma survivor perceives they have failed in the trauma, it can have detrimental future effects for the self. Further, participants reported that a trauma survivor places their faith and trust in their religion. This raises the possibility that some trauma survivors may feel alienated from their religion and their beliefs in their God after having experienced a trauma. Further, individuals reported that trauma survivors often revert to cultural beliefs, rituals and ceremonies to aid in recovery. For instance, P4 brings to light that the Chinese undergo a cleansing ritual every year, namely Chinese New Year. This is a time when they clean their homes and sweep away not just bad luck but any bad experiences they had over the year.

- “for example, in New Year it’s very serious in China. Yeah erm we try to create a cleaning environment so for anything bad, when this is finished, all is returned to normal, everything changed … so it’s, how to say, closure” (P4).

### Future

Participants talked about the future in terms of attitude change and uncertainty appraisals, both of which can impact on adjustment. The trauma can cause a reflection on attitudes concerning life choices and how one pursues their future. Additionally, the trauma event can perpetuate uncertainty appraisals and potentially influences appraisals of future events.

- “I think this is one of the reasons why they are traumatized, because they are worried about their future. So after the trauma they are worried about what could happen to me… they are worried because of the future … They’ll be too much worried about the world for some time” (P2).

#### Education

Although not widely talked about, education did come up as a theme for Focus Groups 1 and 2, whereby they thought it was important for a person to think about what happened to them in order to make sense of the experience and to move on from the trauma. Additionally, participants thought adjusting from the trauma would be harder if education was lacking. For instance, *“I think it [trauma appraisal] would depend on the person and also if they are educated … [because if not educated] the mind would be weak”* (P5). This is also reflective of the research on PTSD susceptibility and trauma recovery (Ahmed 2007).

#### Language

Language was brought up by all key informant interviews. For instance, K1 states *“the “we” is more important than the “I” and if; I speak Turkish and Kurdish and certainly the Kurdish people are speaking to each other in either of those languages, the words they use are “we”, “us” and “our” … you very rarely hear anyone say “I”, “me”, … it can be quite unusual to hear somebody talking very directly about themselves or me as an individual”* (PC). Subtle differences in languages, or use of colloquialisms could result in changing the interpretation of how trauma experiences are reported; which could subsequently impact on assessment interpretations.

## Discussion

This qualitative study aimed to explore the influence of culture on appraisals of trauma within interdependent/collectivistic cultures. Over the course of the focus groups and key informant interviews a strong number of emergent themes arose that had significant consequences for post-trauma psychological adjustment and recovery. In general, members from collectivistic cultures appraised trauma as a predominantly physical stressor, while some did acknowledge psychological distress. This is reflective of previous research conducted with refugees from non-Western cultures, which has shown trauma survivors from these cultural groups to commonly somatise their symptoms. Indeed, several PTSD criteria, such as somatization, are relatively common among Southeast Asian cultures (Eisenhruch 1991).

The group and one’s interconnectedness with the group was very much emphasized with the self (or traumatized self) as a secondary feature of trauma consequences. If considered in terms of the self, one’s social role was called into question, in particular whether the individual could function as part of the group and retain their role within it, or if the trauma had caused a loss or damage to this role; thereby de-valuing the individual as a member of the group. This displacement and feelings of being outside the group, potentially results in extremely poignant feelings of dejection, as the group or family is the individual’s reason for being in many instances. Thus if one is not an active and reciprocal member of the group, the self is devalued on both an individual and collective level, making the trauma’s impact twofold. This further relates back to Ehlers and Clark’s (2000) model, whereby the perception to self as a capable and valuable person is under threat (see also Jobson 2009)

The self is a major component for Western clinical practices in alleviating negative appraisals and restoring a healthy self-concept. However, here it is found that in order to help restore a healthy self-conception, relatedness needs to be taken into account. Namely, relatedness with one’s groups, at either the family or community level is the overarching factor is self-redefinition and reducing self-blame. To address and redress dysfunctional trauma appraisals of the self, focus may need to be given to alleviating distortions concerning how the self impacted on the needs of others. Thus it appears that there is a public appraisal of the self (i.e. viewing the self as a proponent part of the whole and in relation to one’s roles within that whole), where the self has not only been privately damaged, but also viewed as publically humiliated. These public manifestations of self-failure weigh heavily on the individual, because potentially they can no longer see how they fit into the larger world/community or group, creating a sense of isolation and separateness. As falling away from family and society is one of the most profound facets of PTSD.

Again, there are potential practical applications that can be drawn from the data presented. For instance, to aid in trauma recovery, group therapies may be more effective for those form collectivistic cultures. Indeed, group therapies have been widely used in posttraumatic psychotherapy in Western clinical practices due to its ability to reduce psychological shame and to decrease the sense of alienation and isolation that it brings (Adshead 2000). Additionally, the development of self-help groups has been effective in reducing shame and increasing a sense of self-empowerment, challenging passivity and helplessness (Adshead 2000). Moreover, groups have also been used with narrative exposure therapy, which have been shown to be effective with refugee groups (Hinton et al. 2015). Thus group therapy appears to be tapping into both the supportive and prejudiced attitudes the group places on the individual while either encouraging or circumventing them to aid in posttrauma adjustment.

In addition to relationships and social roles being potentially damaged or changed by the trauma, another prominent theme that emerged was that of cultural appropriateness, expectations, values and norms. For many, trauma appraisals were judged and evaluated according to these cultural standards and how one is expected to act within one’s cultural remit, even when dealing with the trauma and its aftermath. Thus culture appears to color one’s interpretations of the events and thus trauma and what constitutes a trauma is based on a particular community’s traditions, mores and values. Moreover, these cultural predilections are expected to be adhered to and act as a base from which an individual is judged. Thus when cultural or societal norms and values are violated by the trauma, it appears individuals may revert to self-blame (e.g. they could have done something to avert it such as being a better citizen).

Participants reported that in some instances trauma experiences are seen as predestined by fate or God or some other external cause. This is associated with another theme that also appears to be a cultural mechanism for coping with trauma, namely, reverting to religion, prayer and cleansing rituals. These could be in keeping with cultural practices following a trauma, for instance, in some collectivistic cultures keeping these customs is part of what it means to be a good citizen. Thereby, by restoring these beliefs, it may help individuals align views of the self with cultural mores on appropriate behaviors and reaffirming that one did not act outside of them to incur the trauma and could aid recovery. Further, in terms of beliefs, religion, ceremonies and rituals, there is a rich literature, especially on fate attribution by ethnographers and cultural observers (Norenzayan and Lee 2010). However, this domain remains largely overlooked in the psychological literature. Taking a social cognitive approach to examining fate beliefs in an attribution framework, the implications of rituals, ceremonies and religion in trauma recovery models would be advantageous to understanding the cognitive underpinnings of such beliefs and their implications for posttrauma recovery. Additionally, deriving more external attributions for the trauma (e.g., beliefs in fate or God as a type of external locus of control, Bond and Tornatzky 1973), is somewhat contrary to the emphasis of those from individualistic cultures who are more likely to exercise primary control and attempt to control or change their external environment (Chun et al. 2006). Here it appears that participants accepted the situational outcomes, for the better or worse. Other themes were similar to research found in Western populations, such as particular emotions (e.g. guilt, shame) and the notion of self-blame. Much research into PTSD has found anger, guilt, shame, and sadness to be high posttrauma, when appraisals of blame, responsibility, and loss become paramount (Amstadter and Vernon 2009).

It is important to note that the mental health professionals were all British and thus not from a collectivistic culture. Yet, on reviewing key informant interviews, similar themes were found to that found in the focus group sessions, with prominent emphasis on: the value of the group to the individual and trauma and recovery being perceived to be experienced at the group/community level. An important point that did emerge from one of the key informant interviews was the potential usefulness of outside help or influences. Thus while the collective group is intertwined with an individual and influences their recovery, sometimes the group may not be able or willing to help. In such instances, help needs to come from the outside. Specifically, through new ideas, thoughts, values and states, which can be introduced by someone external to the group, community or family and allow for the discovery of new appraisal processes. This could potentially help individuals from sacrificing themselves for the group and in so doing release them from an enduring and continuous cycle of self-blame, guilt and shame.

### Limitations

The limitations of the study are acknowledged, the sample size was small which makes all findings tentative and exploratory. However, data saturation was achieved after the first two focus groups and first two key informant interviews. Further, all participants were relatively young and unemployed students, which could have impacted on findings. Additionally, all cultural groups were grouped together and no individualistic focus groups were used; therefore, direct comparisons concerning the themes that emerged cannot be made. Furthermore, while all focus group members acknowledged having experienced a trauma, we did not clinically assess them for PTSD symptoms or depression. This may have impacted on findings as psychological symptoms of distress may influence the appraisals and meaning of trauma discussed by participants (Ehlers and Clark 2000). This, together with the nature of the sample (i.e. university students), also raises questions regarding generalizability of findings to a clinical sample. Additionally, in addition to the small sample size, the collectivistic focus groups were comprised of members from an array of different countries; countries with very different religions, histories and political ideologies. Therefore, whilst all members were from collectivistic cultures, these other variables may have influenced findings. Thus, further research is needed to further investigate these issues. Furthermore, the discussion questions posed to the focus groups were primarily about trauma in general as opposed to asking direct questions relating to the trauma survivors own unique trauma experience. This approach was adopted due to ethical concerns surrounding asking participants to disclose their trauma experience in a group environment and concerns regarding asking direct questions of this nature outside a clinical setting. However, we do not believe this would have impacted negatively on findings as participants would have drawn upon their own knowledge and experiences of trauma within their culture when answering questions on how trauma may be appraised within their cultural group. Future research could, however, examine how culture may moderate the particular appraisals associated with specific trauma types (e.g., does culture have a different influence on appraisals associated with rape as opposed to motor vehicle accidents?). Despite these limitations, this study is important because it is one of the first qualitative studies investigating the interaction between trauma appraisals and culture.

## Conclusions

Notwithstanding these limitations, this study serves to highlight the relationship between trauma and culture. It supports the assertion that it is an important union that warrants further investigation to arrive at culturally informed and appropriate assessment and treatment for those who have experienced trauma. Whilst the study focused on the individualist-collectivist construct, the findings also highlight the importance of considering how other aspects of culture (e.g., religion, determinism, political and historical ideologies etc.) influence trauma-related appraisals. Further the study provides much needed work on research conducted with non-Western populations (Engelbrecht and Jobson 2014; Jobson and O’Kearney 2009; Markus and Kitayama 1991), providing valuable information and insights regarding trauma appraisals. Indeed, the study underscores the many challenges collectivistic cultures face when having undergone a trauma. The findings provide a better understanding about the health-information needs and concerns of collectivistic cultures, and the ways that trauma survivors from these cultures may appraise traumatic events. At the same time, the study helps illuminate the roles for practitioners and health care settings in better serving the needs of those from collectivistic cultures. For instance, it would appear that meanings attached to trauma from community members from collectivistic cultures are centered round their interconnectedness with their group and are interpreted by their cultural values, expectations and social norms. What is more, these culturally shaped beliefs impact an individual’s and even family’s recovery. The next step is to investigate cultures separately; to better understand cultural factors influencing anxiety disorders resultant from a traumatic event.

## Footnotes

1 Researchers identified suitable mental health practitioners via both professional networks (e.g., UK Psychologists working with Refugees network) and approaching key agencies (e.g., trauma clinics, refugee agencies).
